# Integrated Chemometric and Machine Learning Analysis Identifies Peripheral Biosignatures Distinguishing Major Depressive Disorder from Bipolar Disorder: A Translational Cross-Sectional Study

**DOI:** 10.3390/medicina62050806

**Published:** 2026-04-23

**Authors:** Donatella Coradduzza, Stefania Sedda, Andrea Sanna, Alessandra Matilde Nivoli, Maria Rosaria De Miglio, Ciriaco Carru, Massimiliano Grosso, Serenella Medici

**Affiliations:** 1 Department of Biomedical Sciences, University of Sassari, 07100 Sassari, Italycarru@uniss.it (C.C.); 2 SC Chimica Istituto Zooprofilattico Sperimentale della Sardegna, Via Duca degli Abruzzi, 8, 07100 Sassari, Italy; 3 Department of Medicine, Surgery and Pharmacy, University of Sassari, 07100 Sassari, Italydemiglio@uniss.it (M.R.D.M.); 4 Department of Mechanical, Chemical and Materials Engineering, University of Cagliari, 09124 Cagliari, Italy; massimiliano.grosso@unica.it; 5 Department of Chemical, Physical, Mathematical and Natural Sciences, University of Sassari, 07100 Sassari, Italy; sere@uniss.it

**Keywords:** translational biomarkers, major depressive disorder, bipolar disorder, machine learning, chemometrics, partial least squares discriminant analysis, principal component analysis, peripheral inflammation, metallomics, NLRP3 inflammasome

## Abstract

*Background and Objectives*: Major Depressive Disorder (MDD) and Bipolar Disorder (BD) lack objective molecular stratification despite partial clinical overlap, particularly during depressive phases. This cross-sectional study explored whether coordinated peripheral biomarker patterns could be identified using an integrated multivariate analytical framework. *Materials and Methods*: A total of 151 participants (MDD *n* = 41; BD *n* = 40; HC (healthy controls) *n* = 70) were profiled for 42 blood-derived parameters including composite inflammatory indices, hematological markers, trace elements measured by ICP-MS, and circulating BDNF and NLRP3 quantified by ELISA. Data were analyzed using univariate testing, unsupervised dimensionality reduction (PCA, t-SNE), and supervised classification (PLS-DA with cross-validation and permutation testing). *Results*: Thirty-seven of 42 parameters showed significant inter-group differences (*p* < 0.05). Circulating NLRP3 concentrations were markedly reduced in both psychiatric groups compared with HC. Composite inflammatory indices (NLR, SIRI, SII) were elevated in MDD. Zinc levels were modestly reduced, while manganese levels were increased in psychiatric cohorts. BDNF showed lower concentrations in MDD and higher concentrations in BD relative to HC. Cross-validated PLS-DA classification for psychiatric disorder vs. controls yielded an accuracy of 89.4% (AUC-ROC 0.947), with permutation testing indicating performance above chance. However, the sample-to-variable ratio and exploratory design warrant cautious interpretation. *Conclusions*: Multidomain peripheral biomarker profiling identified coordinated biochemical differences across diagnostic groups. These findings suggest the presence of multidimensional peripheral signatures associated with mood disorders within an exploratory framework.

## 1. Introduction

Mood disorders, including Major Depressive Disorder (MDD) and Bipolar Disorder (BD), represent a major public health challenge due to their high prevalence (approximately 5% and 2%, respectively), chronic course, and substantial impact on functional outcomes and quality of life [1,2,3,4]. Current diagnostic paradigms, codified within DSM-5 and ICD-11 taxonomic frameworks, rely predominantly upon phenomenological symptom aggregation assessed through structured clinical interviews, thus lacking integration of objective molecular indices that might enhance diagnostic precision and facilitate early prodromal intervention, so the differential diagnosis between unipolar and bipolar depression remains complex [5,6,7,8,9]. This diagnostic ambiguity is compounded by considerable syndromic overlap between MDD and BD during depressive episodes, with differential diagnosis frequently requiring longitudinal observation spanning multiple mood cycles—a temporal constraint incompatible with urgent clinical decision-making imperatives and contributing to treatment delays and inappropriate medication exposures [10,11,12]. Recent developments in the field of translational psychiatry have given rise to a growing interest in peripheral biomarker profiling as a means of achieving objective diagnostic adjudication and mechanistic subtyping [13]. This interest is predicated on mounting evidence that mood spectrum disorders manifest a systemic physiological variation that is detectable in readily accessible biological matrices [14,15,16,17].

Over the past two decades, a growing body of literature has described mood disorders as conditions associated with systemic biological alterations, including low-grade inflammation, immune imbalance, and redox dysregulation, rather than exclusively central nervous system–restricted diseases [18,19,20,21,22]. Meta-analyses of existing studies have consistently reported elevated circulating levels of pro-inflammatory mediators in individuals with mood disorders compared with controls [18,23,24,25,26,27]. These findings support the presence of a peripheral inflammatory milieu. However, the directionality and causal relevance remain unclear. From a biological standpoint, several mechanisms have been proposed to explain how peripheral inflammation may interact with central nervous system function [28,29]. These include the modulation of monoaminergic neurotransmission through prostaglandin-mediated pathways, increased permeability of the blood–brain barrier facilitating immune cell trafficking, and microglial priming leading to amplification of neuroinflammatory signaling [30,31]. However, most available evidence derives from experimental or translational models, and direct mechanistic confirmation in clinical populations remains limited. Peripheral inflammatory processes have been consistently reported in subsets of patients with mood disorders and have been associated with symptom severity, illness chronicity, and treatment response [32,33,34]. In this context, blood-based biomarkers represent a practical and accessible means of exploring disease-associated biological patterns. Complementary investigations have extended this conceptual framework to composite hematological inflammatory indices derived from routine laboratory parameters, including the neutrophil-to-lymphocyte ratio (NLR), systemic inflammatory response index (SIRI), and systemic immune-inflammation index (SII) [35,36,37]. These indices integrate the relative distribution of leukocyte subpopulations into quantitative metrics that are considered proxies of systemic inflammatory balance. Compared with isolated cell counts, such composite indices may provide a more stable representation of inflammatory tone, although they remain non-specific and must be interpreted within a broader clinical and biological context [38,39,40,41].

In parallel, dysregulated trace element homeostasis has been implicated in mood disorder etiology, wherein essential micronutrients (e.g., zinc, selenium) and potentially toxic metalloids (e.g., arsenic, cadmium, lead) may modulate neurotransmitter synthesis, antioxidant defense systems, and mitochondrial bioenergetics through pleiotropic molecular mechanisms [42,43,44].

However, interpretation of peripheral trace element concentrations is complicated by multiple confounding factors, including dietary variability, diurnal rhythms (with zinc exhibiting approximately 15% circadian fluctuation between morning and evening measurements), preanalytical variables (hemolysis can artificially elevate zinc by 15–20% due to erythrocyte lysis), and individual metabolic handling differences. Furthermore, peripheral metal concentrations reflect complex interactions between environmental exposure, nutritional intake, metabolic handling, and blood–brain barrier transport and therefore should be interpreted as contextual biological indicators rather than direct etiological determinants [45,46,47].

Furthermore, molecular mediators including brain-derived neurotrophic factor (BDNF) and NLRP3 inflammasome components have been posited as critical regulatory nodes governing neuroplasticity and innate immune sensing, respectively, with hypothesized perturbations in mood psychopathology [15,16]. However, prior biomarker investigations have typically employed isolated univariate statistical comparisons of individual analytes, thus precluding detection of coordinate multivariate biosignature patterns that may exhibit superior discriminatory capacity through synergistic integration of orthogonal measurement domains [47,48,49,50,51].

Emerging evidence further supports a multidomain biological framework in which trace element imbalance, neurotrophic dysregulation, and innate immune activation may converge in subsets of patients with mood disorders. Essential elements, including selenium, zinc, copper and iron, are involved in antioxidant defense systems, thyroid hormone metabolism, mitochondrial bioenergetics, and monoaminergic neurotransmitter synthesis. Conversely, exposure to heavy metals such as lead and cadmium has been associated in epidemiological cohorts with increased risk of depressive and anxiety disorders, potentially through mechanisms involving mitochondrial dysfunction and reactive oxygen species generation [52,53,54,55,56].

In parallel, converging meta-analytic evidence indicates reduced peripheral levels of brain-derived neurotrophic factor during both depressive and manic episodes, with partial normalization during euthymia. BDNF plays a central role in synaptic plasticity and neurogenesis, and experimental data suggest that pro-inflammatory cytokines derived from inflammasome activation may suppress BDNF expression, thereby linking immune signaling to neurotrophic modulation. Large meta-analyses have consistently reported alterations in inflammatory markers and BDNF levels in mood disorders, although with substantial heterogeneity and limited diagnostic specificity [18,57,58,59].

The NLRP3 inflammasome, a key sensor of cellular stress and mitochondrial dysfunction, has been reported to exhibit increased gene expression and functional activation in peripheral blood mononuclear cells of patients with mood disorders, although discrepancies between transcriptomic, protein, and circulating measurements [60,61,62,63] underscore the need for cautious interpretation [58,64,65,66,67,68].

Emerging experimental evidence further suggests a bidirectional regulatory relationship between BDNF signaling and NLRP3 inflammasome activity [69]. BDNF–TrkB activation has been shown to suppress NLRP3-mediated pyroptotic pathways and inflammatory cytokine release, while conversely, upstream inflammatory mediators associated with NLRP3 activation (e.g., IL-1β, TNF-α) may impair BDNF signaling and synaptic plasticity [70]. This reciprocal interaction has been described across multiple biological systems, including vascular, neurodegenerative, and affective disorder models, and provides a biologically plausible framework for the concurrent alterations observed in these markers [71,72].

Taken together, these observations suggest that alterations in metallomic homeostasis, neurotrophic support, and innate immune signaling may co-occur within a shared redox–inflammatory framework. Nevertheless, most studies have evaluated these biological domains in isolation using univariate analytical approaches, limiting the capacity to identify integrated biosignature patterns that may better capture the systemic biological heterogeneity underlying mood disorders [18,63,73,74].

The present study was designed as an observational, cross-sectional investigation aimed at characterizing peripheral biological variation across diagnostic groups using a structured statistical framework. The analytical strategy included univariate evaluation of 42 blood-derived biomarkers encompassing inflammatory indices, standard hematochemical parameters, trace and toxic metals, and selected inflammation-related molecular markers. In addition, unsupervised multivariate approaches, including Principal Component Analysis (PCA) and t-distributed Stochastic Neighbor Embedding (t-SNE), were applied to explore intrinsic variance structure without imposing predefined class separation. Supervised modeling using Partial Least Squares Discriminant Analysis (PLS-DA) was performed to examine multivariate separation patterns, and Variable Importance in Projection (VIP) scores were calculated to quantify the relative contribution of individual features within the model [75,76]. This approach was not intended to establish diagnostic accuracy, predictive validity, or mechanistic inference. Rather, the objective was to determine whether coordinated patterns of peripheral biomarker variation could be statistically identified within a multivariate framework. Given the cross-sectional design, all findings should be interpreted as associative and exploratory [77]. Any potential biological or clinical implications require independent replication and longitudinal validation before consideration of translational relevance.

## 2. Materials and Methods

### 2.1. Study Design and Population

This study was conducted as an observational, cross-sectional investigation designed to characterize peripheral inflammatory, hematochemical, metal-related, and molecular signatures in mood disorders. Participants were consecutively recruited at the Psychiatry Unit of the Azienda Ospedaliero-Universitaria of Sassari, in collaboration with the Department of Biomedical Sciences, University of Sassari. The study population consisted of three groups of adult subjects aged ≥18 years, with no upper age limit applied: (i) patients diagnosed with Major Depressive Disorder (MDD); (ii) patients with Bipolar Disorder (BD), including type I and type II, evaluated during euthymic or mildly symptomatic phases; and (iii) Healthy Controls (HC) with no personal or family history of psychiatric disorders. Psychiatric diagnoses were established by trained psychiatrists according to the Diagnostic and Statistical Manual of Mental Disorders, Fifth Edition, Text Revision (DSM-5-TR), using the Structured Clinical Interview for DSM-5 Disorders (SCID-5). Symptom severity was assessed using the 17-item Hamilton Depression Rating Scale (HDRS-17) to evaluate depressive symptoms and the Young Mania Rating Scale (YMRS) to assess manic symptoms [77]. Inclusion criteria were age ≥ 18 years, a confirmed psychiatric diagnosis for the patient groups, and the ability to provide written informed consent. Exclusion criteria included the presence of acute or chronic inflammatory, autoimmune, infectious, metabolic, or endocrine diseases; pregnancy or breastfeeding; substance or alcohol abuse within the previous six months; and chronic treatment with corticosteroids or immunomodulatory agents. Participants with clinical evidence of acute infection at time of sampling (assessed via clinical interview) or elevated inflammatory markers (C-reactive protein > 10 mg/L) were excluded to minimize acute phase response confounding. Mood disorder cohorts received naturalistic pharmacological treatment reflective of real-world clinical practice.

### 2.2. Ethical Approval

The study was conducted in accordance with the Declaration of Helsinki and approved by the Fondazione Policlinico Universitario A. Gemelli IRCCS—Università Cattolica del Sacro Cuore (Protocol n. 0020985/22). The study received favorable opinion on 28 July 2022. All participants received detailed information about the study procedures and provided written informed consent prior to enrollment. Data were anonymized in compliance with Regulation (EU) 2016/679 (GDPR).

### 2.3. Blood Sampling and Pre-Analytical Procedures

Peripheral venous blood specimens were collected following overnight fasting (≥8 h) with standardized morning timing (08:00–10:00). Blood was drawn into EDTA-containing tubes and processed within 30 min of collection. Samples were centrifuged at 1500× *g* rpm for 10 min at 4 °C. Plasma was aliquoted into cryovials pre-chilled on dry ice and stored at −80 °C with single freeze–thaw cycle restriction until analysis. All samples were processed under standardized conditions and analyzed in a blind manner. Haemolysis was assessed visually and by spectrophotometric index prior to analysis; samples with hemolysis index > 2+ were excluded from iron quantification due to the risk of artificially elevated values from erythrocyte lysis (see Table 1).

### 2.4. Determination of Plasma NLRP3 and BDNF Levels

Plasma levels of human NLRP3 were measured using a sandwich ELISA kit (ELK Biotechnology, Wuhan, China, Catalog #ELK1496), according to the manufacturer’s instructions. Briefly, 100 µL of standards and plasma samples were assayed in duplicate, and concentrations were calculated from a four-parameter logistic standard curve (r^2^ > 0.995). Assay validation metrics: intra-assay coefficient of variation (CV) 6.8%, inter-assay CV 11.3%, detection range 0.156–10 ng/mL (156–10,000 pg/mL), limit of quantification (LOQ) 0.156 ng/mL (156 pg/mL). Samples from HC participants with concentrations exceeding the kit upper detection limit (10 ng/mL) were re-analysed after serial dilution (1:20 and 1:50) in assay diluent; reported values represent back-calculated concentrations. A total of 43 out of 70 HC samples (61.4%) required dilution; all diluted samples met the intra-duplicate CV acceptance criterion (<12%). The higher observed standard deviation in the HC group reflects the additional analytical uncertainty inherent in back-calculated values at extended dilution factors, and is acknowledged as a limitation of the assay in this concentration range.

Plasma BDNF concentrations were determined using a human BDNF ELISA kit (RayBiotech, Peachtree Corners, GA, USA, Catalog #ELH-BDNF). Following the manufacturer’s protocol, 100 µL of standards and samples was analyzed in duplicate, and mean values were used for statistical analysis. Assay validation metrics: intra-assay CV 4.8%, inter-assay CV 9.1%, detection range 31.2–2000 pg/mL, LOQ 31.2 pg/mL. Samples below LOQ were assigned the LOQ value for statistical analysis.

Quality assurance procedures for both assays included: (i) duplicate analysis of all samples with acceptance criterion of intra-duplicate CV < 15%; (ii) inclusion of positive and negative controls on each plate; (iii) single freeze–thaw cycle restriction for all samples; (iv) processing of samples from all diagnostic groups in randomized batch order to minimize systematic bias. Samples with concentrations below the LOQ were assigned the LOQ value for statistical analysis. All ELISA procedures were performed by trained personnel blinded to diagnostic group assignment.

### 2.5. Plasma Metals and Metalloids Analysis Using ICP-MS

Total metals and metalloids in plasma (aluminium, antimony, arsenic, barium, beryllium, cadmium, chromium, cobalt, copper, iron, lead, lithium, manganese, mercury, nickel, molybdenum, selenium, silver, thallium, tin, vanadium and zinc) were determined by inductively coupled plasma mass spectrometry (ICP-MS) in accordance with ISO 17294-2:2016 [78,79], supplemented by the laboratory’s validated standard operating procedure accredited under UNI EN ISO 17025:201726 [80]. Biological fluids were analysed directly after dilution of 0.5 mL of sample in 5 mL with 2% nitric acid (J.T. Baker, Phillipsburg, NJ, USA) solution. The analysis was performed with an inductively coupled plasma mass spectrometer ICP-MS/MS (Agilent Technologies, Santa Clara, CA, USA) equipped with a collision cell and two quadrupole mass analyzers. In comparison to a single quadrupole ICP-MS system, the triple quadrupole system significantly increases the accuracy of mass separation. To compensate for the matrix effect and signal drift, a solution of internal standards was used. The calibration curve was verified at the start of each analytical batch using the initial calibration verification (ICV) with a different lot standard, while the instrumental sensitivity was verified using the continuous calibration verification (CCV) at or near midrange. The LOQs testing was 0.001 ng/mL for all elements analysed. The quality control of the data was verified and controlled using Certified Reference Materials ClinChek^®^ Plasma Control for Trace Elements, (RECIPE Chemicals, Munich, Germany). Laboratory was intercalibrated through successful participation in internationally organized proficiency tests (OELM). The method is accredited according to UNI EN ISO 17025/2017 [80].

### 2.6. Statistical Analysis

#### 2.6.1. Univariate Statistical Analysis

Distributional normality was assessed using the Shapiro–Wilk test (α = 0.05). Normally distributed variables were analyzed using one-way ANOVA, while non-parametric variables were assessed using the Kruskal–Wallis H test.

In the initial exploratory phase, analyses were conducted without correction for multiple comparisons to prioritize sensitivity. However, in the final analytical pipeline, all univariate comparisons were adjusted using the Benjamini–Hochberg false discovery rate (FDR) procedure, and corresponding q-values are reported.

Statistical significance was defined as two-tailed *p* < 0.05. All analyses were performed using R version 4.5.0 (R Foundation for Statistical Computing, Vienna, Austria).

#### 2.6.2. Multivariate Statistical Analysis—Unsupervised Methods

Unsupervised and supervised machine learning methodologies were employed to overcome the limitations of univariate analytical approaches and to identify coordinated multidimensional biosignature patterns [81]. Prior to multivariate modeling, the data matrix (151 observations × 42 variables) underwent preprocessing including: autoscaling (mean-centering and unit variance scaling) to equalize variable contributions irrespective of native measurement scales [82]. Observations exceeding this threshold were considered statistically inconsistent with the overall data structure and were therefore examined prior to model construction [83,84,85,86].

Principal Component Analysis (PCA) was performed as the main unsupervised linear dimensionality reduction method to explore the underlying variance structure of the dataset and to evaluate whether samples exhibited natural clustering patterns, without using any prior diagnostic class labels. This approach provides a first-order approximation of the data structure in a linear subspace. PCA decomposes the multivariate data matrix into orthogonal linear combinations (principal components) capturing maximal variance in descending order, with eigenvalue > 1 criterion and cumulative explained variance ≥ 70% employed for component retention [23]. Score plots visualize sample distribution in reduced-dimensional space, while loading plots identify variables contributing most substantially to each component.

While PCA provides a linear approximation of the data structure, it may not fully capture more complex, non-linear relationships between variables. The t-distributed Stochastic Neighbor Embedding (t-SNE) technique is therefore employed as a complementary non-linear technique, specifically designed to preserve local similarities between observations and to enhance the visualisation of potential clustering patterns. Unlike linear methods such as PCA, t-SNE is able to capture more complex relationships in the data while preserving the similarity between neighboring observations [87]. Reducing high-dimensional data to two- or three-dimensional representations facilitates intuitive visual exploration and interpretation of the underlying structure.

Overall, the combined use of linear and non-linear dimensionality reduction techniques allows a more comprehensive characterization of the underlying data structure.

#### 2.6.3. Multivariate Statistical Analysis—Supervised Methods

In contrast to the unsupervised methods described above, which do not use any prior information on group membership, a supervised approach was employed to directly relate biomarker profiles to diagnostic categories. For this purpose, Partial Least Squares Discriminant Analysis (PLS-DA) was applied. PLS-DA constructs regression models maximizing covariance between predictor variables (biomarker panel) and categorical response variables (diagnostic class membership). PLS-DA extends classical PLS regression to categorical outcomes through dummy variable encoding, identifying latent variable combinations optimally discriminating between groups while simultaneously managing multicollinearity inherent in high-dimensional biomarker datasets [88,89]. Model construction employed systematic evaluation of 1–10 latent variables, with optimal dimensionality selected via minimization of root mean square error of cross-validation (RMSECV) [90,91]. Stratified 5-fold cross-validation with 100 repetitions assessed predictive performance through calculation of accuracy, sensitivity, specificity. Variable Importance in Projection (VIP) scores quantified the contribution of each biomarker to model discrimination, with VIP > 1.0 conventionally indicating substantial importance and VIP > 2.0 denoting exceptional discriminatory capacity [92]. Within this framework, PLS-DA was primarily used as a feature-ranking and dimensionality reduction tool (via VIP scores), rather than as a predictive modeling approach.

## 3. Results

### 3.1. Demographic, Hematological and Biosignature Profiling

The study cohort included 151 participants distributed across three diagnostic groups (Table 2). All univariate comparisons were corrected for multiple testing using the Benjamini–Hochberg false discovery rate (FDR) procedure; q-values are reported alongside *p*-values in Table 3, Table 4, Table 5, Table 6 and Table 7. Significant differences were observed in sex distribution across groups (χ^2^ = 70.65, *p* < 0.0001), with a higher proportion of females in the MDD group and a predominance of males in the BD group. Age also differed between diagnostic categories. One-way ANOVA indicated a significant group effect (F = 3.26, *p* = 0.041), which was confirmed by the Kruskal–Wallis test (H = 10.99, *p* = 0.004) (Table 2). On average, participants with BD were younger than those with MDD and HC.

**Table 1:** Demographic Characteristics of the Study Population.

Group	N	Female (n)	Male (n)	Age, Mean ± SD (Years)	Age Range (Years)
HC	70	45	25	57.04 ± 18.89	20–87
MDD	41	34	7	59.44 ± 10.00	31–81
BD	40	17	23	51.48 ± 8.09	29–63
Total Sample	151	96	55	56.22 ± 14.73	20–87

**Table 2:** Statistical comparison of demographic variables across diagnostic groups.

Variable	Test Statistic	*p*-Value
Sex distribution	χ^2^ = 70.65	*p* < 0.001
Age	F = 3.26	*p* = 0.041
Age	H = 10.99	*p* = 0.004

**Table 3:** Plasma BDNF concentrations across diagnostic groups and post-hoc pairwise comparisons.

(A) Descriptive statistics							
**Group**	**n**	**Mean (ng/mL)**	**SD**	**Median**	**IQR**	**Min**	**Max**
HC	70	0.522	0.347	0.476	0.586	0.050	1.256
MDD	41	0.136	0.036	0.128	0.050	0.096	0.200
BD	40	0.629	0.052	0.630	0.082	0.540	0.720
(B) Post-hoc pairwise comparisons							
**Comparison**	**U Statistic**	***p*** **-Value**	**Effect Size (r)**	**Significance**			
HC vs. MDD	2422	<0.000001	−0.688	***			
HC vs. BD	1114	0.076	0.204	ns			
MDD vs. BD	0	<0.000001	1.000	***			

Statistical significance: *** *p* < 0.001; ns: not significant.

**Table 4:** Plasma NLRP3 concentrations across diagnostic groups.

Group	N	Mean	SD	Min	Q1	Median	Q3	Max	IQR
HC	70	122.43	22.10	79.34	106.51	120.77	136.24	180.47	29.89
MDD	41	2.68	0.22	2.18	2.50	2.70	2.90	3.10	0.40
BD	40	0.88	0.13	0.63	0.79	0.90	0.98	1.12	0.19

**Kruskal–Wallis test:** H = 129.29, *p* < 0.000001.

**Table 5:** Composite Inflammatory Indices Across Diagnostic Groups.

Index	Group	N	Mean ± SD	Median	IQR	*p*-Value
**NLR**	HC	70	1.62 ± 0.83	1.51	0.51	*p* < 0.001
	MDD	41	2.95 ± 1.11	2.69	0.92	
	BD	40	1.86 ± 0.63	1.71	0.64	
**MLR**	HC	70	0.21 ± 0.09	0.20	0.12	*p* < 0.001
	MDD	41	0.33 ± 0.09	0.31	0.12	
	BD	40	0.23 ± 0.05	0.22	0.07	
**PLR**	HC	70	104.53 ± 37.50	93.49	49.25	*p* < 0.001
	MDD	41	136.94 ± 36.01	136.69	33.95	
	BD	40	102.70 ± 32.34	99.72	46.82	
**SIRI**	HC	70	0.77 ± 0.57	0.60	0.68	*p* < 0.001
	MDD	41	1.66 ± 0.70	1.62	0.82	
	BD	40	1.02 ± 0.37	0.93	0.42	
**SII**	HC	70	357.66 ± 190.61	314.65	139.96	*p* < 0.001
	MDD	41	668.35 ± 260.62	639.18	186.66	
	BD	40	469.80 ± 236.25	410.91	222.39	

**Table 6:** Hematological parameters across diagnostic groups.

Parameter	Unit	HC (Mean ± SD)	MDD (Mean ± SD)	BD (Mean ± SD)	*p*-Value
WBC	×10^3^/μL	6.33 ± 1.66	7.56 ± 1.28	7.71 ± 1.05	<0.001
NEU	×10^3^/μL	3.60 ± 1.45	4.89 ± 1.20	4.46 ± 1.07	<0.001
LYM	×10^3^/μL	2.27 ± 0.59	1.74 ± 0.35	2.51 ± 0.50	<0.001
MONO	×10^3^/μL	0.45 ± 0.17	0.56 ± 0.07	0.55 ± 0.08	<0.001
EOS	×10^3^/μL	0.14 ± 0.09	0.11 ± 0.06	0.17 ± 0.04	<0.001
BASO	×10^3^/μL	0.05 ± 0.04	0.07 ± 0.05	0.05 ± 0.03	0.0469
RBC	×10^6^/μL	4.72 ± 0.54	4.82 ± 0.39	5.04 ± 0.22	<0.001
HGB	g/dL	13.46 ± 1.12	13.75 ± 1.34	14.86 ± 0.96	<0.001
MCV	fL	83.28 ± 7.62	87.94 ± 4.67	87.37 ± 2.49	<0.001
MCH	pg	28.39 ± 3.04	30.39 ± 1.89	30.60 ± 1.42	<0.001
MCHC	g/dL	34.62 ± 0.72	34.68 ± 0.59	34.89 ± 0.69	0.2348
RDW	%	12.87 ± 0.64	13.76 ± 0.98	13.56 ± 0.59	<0.001
PLT	×10^3^/μL	221.26 ± 46.64	229.95 ± 42.83	247.15 ± 56.11	0.0631
MPV	fL	11.39 ± 0.80	10.78 ± 1.13	10.89 ± 1.03	0.0079

**Table 7:** Plasma trace elements across diagnostic groups.

Metal	Unit	HC (Mean ± SD)	MDD (Mean ± SD)	BD (Mean ± SD)	*p*-Value
Iron	μg/L	1019.84 ± 313.74	1104.06 ± 206.99	—*	0.1437
Zinc	μg/L	1295.14 ± 228.54	1171.88 ± 184.27	1154.19 ± 160.24	0.0027
Copper	μg/L	1128.82 ± 406.76	1029.62 ± 218.16	1007.85 ± 217.87	0.2973
Selenium	μg/L	114.69 ± 19.58	121.91 ± 18.39	114.76 ± 13.06	0.1039
Manganese	μg/L	1.76 ± 0.47	2.13 ± 0.34	2.61 ± 0.46	<0.001
Cobalt	μg/L	0.37 ± 0.13	0.44 ± 0.08	0.41 ± 0.07	<0.001
Lithium	μg/L	3.20 ± 2.99	1412.63 ± 2854.09	976.98 ± 2165.31	<0.001
Chromium	μg/L	0.42 ± 0.77	1.40 ± 0.31	1.44 ± 0.37	<0.001
Nickel	μg/L	0.15 ± 0.33	1.20 ± 1.02	0.54 ± 0.78	<0.001
Arsenic	μg/L	3.13 ± 5.76	3.80 ± 3.06	2.16 ± 0.55	<0.001
Molybdenum	μg/L	0.65 ± 0.34	3.46 ± 10.17	1.07 ± 0.32	<0.001
Cadmium	μg/L	0.04 ± 0.07	0.06 ± 0.05	0.02 ± 0.04	<0.001
Lead	μg/L	0.24 ± 0.35	0.23 ± 0.06	0.18 ± 0.06	0.0043
Mercury	μg/L	1.05 ± 0.88	0.58 ± 0.23	0.73 ± 0.60	0.0074
Beryllium	μg/L	0.04 ± 0.08	0.07 ± 0.06	0.03 ± 0.03	0.0027

—*: Iron values for the BD group indicates data not available due to exclusion of hemolyzed samples. Iron values for the BD group were not reported for this reason, as specified in Section 2.3.

#### 3.1.1. Neurotrophic and Inflammasome Markers

Significant differences were observed for both BDNF and NLRP3 across diagnostic groups (*p* < 0.001). BDNF concentrations were reduced in MDD and increased in BD relative to healthy controls, whereas NLRP3 levels were markedly lower in both psychiatric groups (see Table 3 and Table 4). Age and sex were included as covariates in ANCOVA models for primary biomarkers; the main group effects remained significant after adjustment (see Supplementary Table S1).

#### 3.1.2. Inflammatory Indices

All composite inflammatory indices showed significant inter-group differences (*p* < 0.001). A consistent pattern of elevation was observed in MDD compared with both BD and healthy controls. Pairwise analyses confirmed that MDD differed significantly from HC and BD across all indices. In contrast, BD showed only partial differences compared with controls, with significant changes observed for NLR, SIRI, and SII, while MLR and PLR remained comparable (see Table 5).

#### 3.1.3. Hematological Parameters

Several hematological parameters differed significantly across groups, particularly those related to leukocyte distribution and erythrocyte indices (*p* < 0.05). In contrast, mean corpuscular hemoglobin concentration (MCHC) and platelet count (PLT) did not show significant differences (see Table 6).

#### 3.1.4. Trace Elements

Significant inter-group differences were observed for selected essential trace elements, particularly zinc, manganese, and cobalt, while others (including iron, copper, and selenium) did not differ significantly. Several potentially toxic elements also showed marked variation across groups, including lithium, chromium, nickel, arsenic, molybdenum, cadmium, lead, mercury, and beryllium (see Table 7).

### 3.2. Multivariate Statistical Analysis

#### 3.2.1. Unsupervised Analysis—Principal Component Analysis

**Figure 1:**
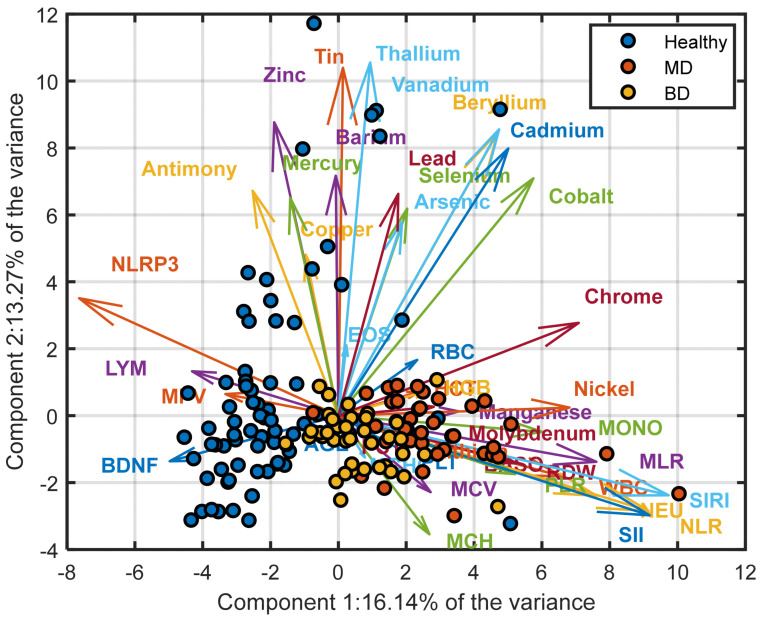
Principal Component Analysis (PCA) biplot (PC1 vs. PC2) of the autoscaled 42-biomarker dataset. Points represent individual subjects colored by diagnostic group (HC, MDD, BD), and arrows indicate variable loadings. PCA was performed as an exploratory, unsupervised analysis to visualize variance structure without class labels.

**Figure 2:**
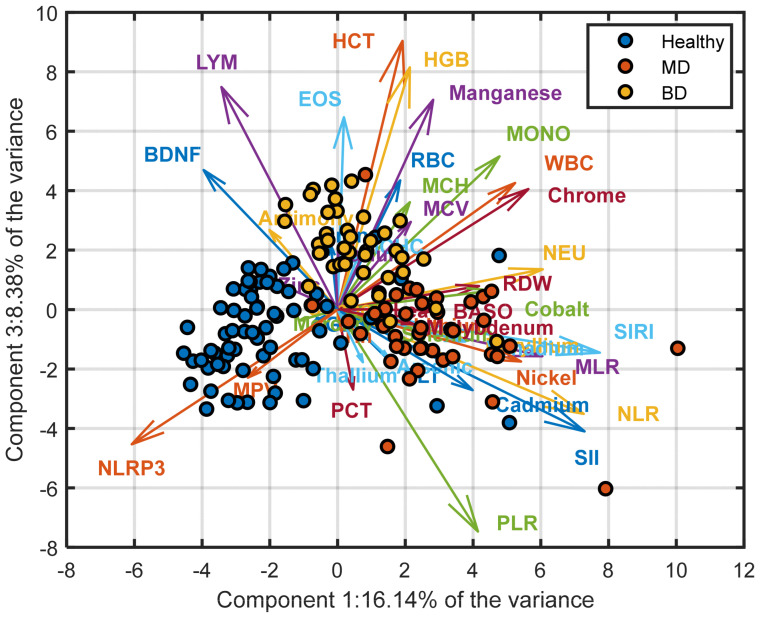
PCA biplot (PC1 vs. PC3) illustrating additional orthogonal variance structure. PC3 captures 8.38% of total variance and highlights contributions from erythrocyte indices, BDNF, and haematological parameters, supporting multidimensional biosignature architecture.

PCA of the autoscaled 42-biomarker dataset identified 12 components with eigenvalues > 1, explaining 78.3% of the total variance. The first three principal components accounted for 55.3% of the variance (PC1: 28.4%, PC2: 15.7%, PC3: 11.2%). The PC1–PC2 projection (Figure 1) showed a structured distribution of samples, with healthy controls forming a relatively compact cluster, MDD patients predominantly located at negative PC1 values, and BD individuals occupying intermediate positions with partial overlap between groups. The scree plot illustrating the variance explained by each principal component is provided in Supplementary Figure S1. Differences among diagnostic categories were primarily reflected along PC1, with separation of the MDD class, suggesting that disease status may contribute to the main axis of variation. Inspection of the loading patterns indicated that this separation reflects a contrast between inflammatory indices and selected hematological and metallomic variables. Higher values of NLR, NEU, and SIRI were associated with the MDD cluster, whereas higher lymphocyte counts and NLRP3 levels were more characteristic of the healthy group. The PC1–PC3 projection (Figure 2) further highlighted differences between BD and the other groups. In this representation, BD samples showed relatively higher scores along PC3, suggesting an additional dimension of variability potentially related to erythrocyte indices and trace elements. Overall, PCA supports the presence of structured multivariate variation across diagnostic groups, while the observed overlap indicates that these patterns reflect biological heterogeneity rather than discrete class separation.

Figure 2 presents the PCA biplot for the first and third principal components. Compared with the PC1–PC2 projection, the distinction of the MDD class from the other two groups appears more pronounced in this representation. In particular, BD individuals are characterized by relatively higher scores along with the third principal component. This pattern suggests that PC3 captures variability that contributes to the differentiation of the BD class from both HC and MDD patients. Inspection of the loading vectors provides further insight into this structure. First, NLRP3 consistently distinguishes between healthy and diseased groups, with higher NLRP3 values in the control sample. Second, NLR, SIRI, and Cadmium load in the direction of the MDD cluster, indicating that elevated levels of these markers are characteristic of the MDD class within this projection. Third, EOS, HCT, HGB, and Manganese display positive loadings along the third principal component, suggesting that higher values of these variables are associated with the BD group and may underline its separation along PC3.

#### 3.2.2. Unsupervised Analysis—t-SNE

Although principal component analysis suggested group-related structure, a relatively large number of components were required to account for most of the total variance. This indicates that the data’s underlying structure spans multiple linear dimensions and may not be efficiently summarized in a low-dimensional linear subspace. To further investigate whether class differences could be represented more parsimoniously, we applied t-SNE, a non-linear dimensionality reduction technique designed to preserve local neighborhood relationships [93]. Raw data were first preprocessed using PCA, and the first 30 principal components, accounting for 95% of the total variance, were retained. The hyperparameters were set to a perplexity of 10, an exaggeration factor of 4, and a learning rate of 500; the random number generator was initialized using the first seed in Matlab^®^ (MathWorks, Natick, MA, USA; version R2023a). Cosine distance was used as the distance metric. The resulting two-dimensional embeddings demonstrated spatial organization consistent with group differences, with visual separation between diagnostic categories. It should be noted that t-SNE, by design, optimizes local neighborhood preservation and may exaggerate the apparent degree of cluster separation as an artefact of its cost function (KL divergence minimization); the spatial organization observed in the embedding should therefore be interpreted as suggestive of structural differences rather than proof of discrete biological categories. Comparable hyperparameter configurations yielded similar embedding structures. HC samples occupied a distinct region with limited spatial overlap with the psychiatric cohorts, apart from two observations located within the BD region. MDD and BD samples showed substantial spatial separation, with limited overlap, a pattern spatially consistent with the presence of both shared and diagnosis-specific biomarker variation.

**Figure 3:**
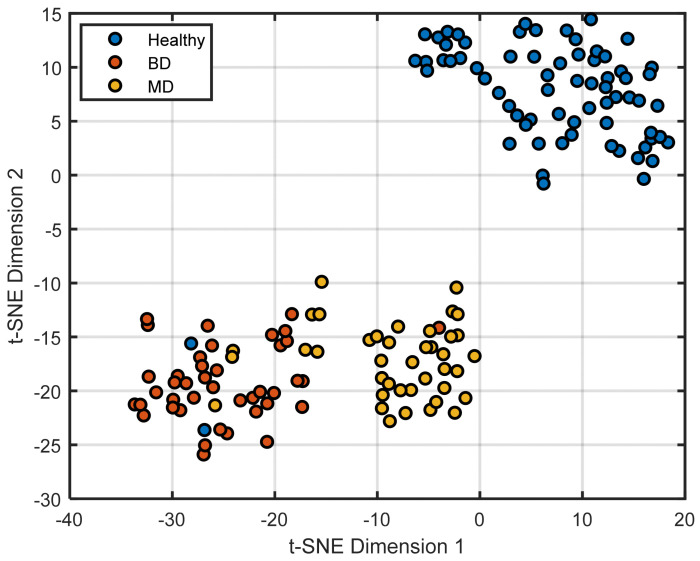
Two-dimensional t-SNE embedding of the autoscaled dataset (perplexity = 10, exaggeration = 4, learning rate = 500). Points represent individual observations colored by class (HC, BD, MDD). t-SNE was applied as an exploratory, non-linear dimensionality reduction technique to visualize local data structure; axes do not correspond to interpretable linear combinations of variables.

The convergence of linear (PCA) and non-linear (t-SNE) unsupervised approaches in revealing spatially consistent group-related organisation provides complementary evidence that the observed biomarker variation is not attributable to isolated analyte fluctuations or technical artefacts. However, the degree of visual separation observed—particularly in the t-SNE embedding—should be interpreted cautiously, as both methods can amplify apparent structure in ways that do not map directly onto biological discreteness, Figure 3.

#### 3.2.3. Supervised Analysis—Partial Least Squares Discriminant Analysis

**Figure 4:**
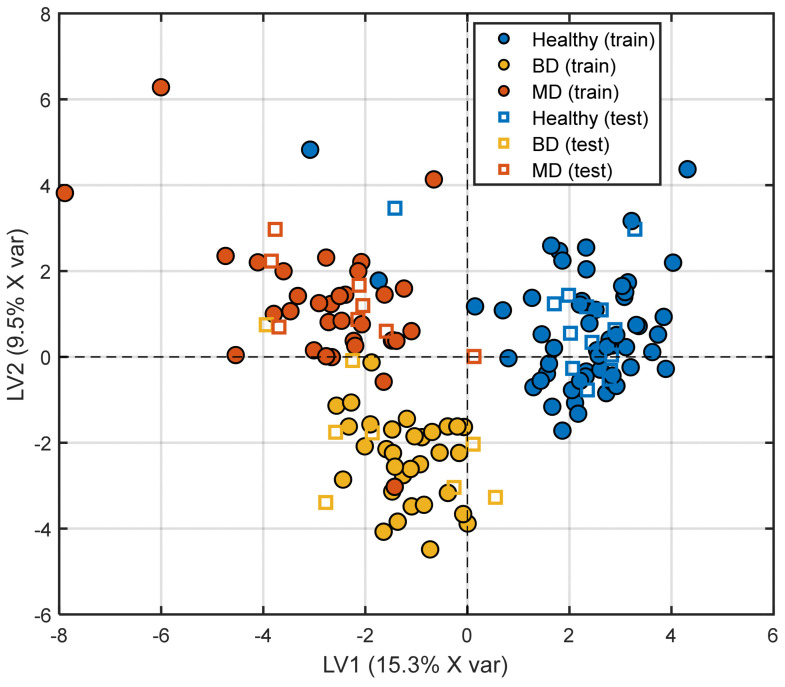
Partial Least Squares Discriminant Analysis (PLS-DA) score plot (LV1 vs. LV2) based on the autoscaled 42-biomarker dataset. Training samples are shown as filled circles, whereas internal hold-out validation samples are represented by open squares. Colors indicate class membership: MD (orange), BD (yellow), and HC (blue). The percentage of explained variance is reported on each axis.

The PLS-DA model was constructed using two latent variables and applied to an autoscaled dataset comprising 42 biomarkers. Model performance was evaluated by means of stratified five-fold cross-validation, following the procedure described in Section 2.6.2. The dataset was partitioned into a training set (*n* = 121; HC = 56, BD = 32, MD = 33) and an Internal hold-out validation set (*n* = 30; HC = 14, BD = 8, MD = 8). The score plot (Figure 4) demonstrated clear separation among diagnostic groups, with limited overlap between BD and MDD samples and test set samples projecting within the same regions as the corresponding training classes. The model explained 24.8% of the X variance (R^2^X = 0.248) and 44.0% of the Y variance (R^2^Y = 0.440) with two latent variables. The cross-validated classification accuracy on the training set was 96.5% (Q^2^Y = 0.054). Internal hold-out validation on the held-out test set (*n* = 30) yielded an accuracy of 89.3% on assigned samples (28/30), with misclassifications occurring primarily at the BD/MDD boundary, consistent with the known clinical overlap between these conditions. Permutation testing (999 permutations) confirmed that the model’s discriminant ability was not attributable to chance (R^2^Y *p* < 0.001; Q^2^Y *p* < 0.001; (see Supplementary Figure S2). Overall, these results indicate that multivariate integration of peripheral biomarkers captures structured variation within the dataset; however, classification performance should be considered descriptive rather than indicative of generalizable predictive accuracy.

Table 8 reports the confusion matrix and performance metrics for the PLS-DA classification. The training-set accuracy was 96.5% (error rate 3.4%), with high per-class metrics across all groups (HC: sensitivity 0.964, precision 1.000; BD: sensitivity 0.966, precision 0.966; MDD: sensitivity 0.968, precision 0.909). Misclassifications were sparse and concentrated at the BD/MDD boundary, and 6 samples remained unassigned. The cross-validated Q^2^Y (0.054) provides a more conservative estimate of predictive ability, reflecting the degree of model optimism inherent in the sample-to-variable ratio of this dataset. Table 8 and Table 9 report the confusion matrices and performance metrics for the PLS-DA model on the training and external validation sets. The training-set accuracy was 96.5% (error rate 3.4%), with high per-class metrics across all groups (HC: sensitivity 0.964, precision 1.000; BD: sensitivity 0.966, precision 0.966; MDD: sensitivity 0.968, precision 0.909) and sparse misclassifications concentrated at the BD/MDD boundary. The cross-validated Q^2^Y (0.054) reflects the expected degree of model optimism given the sample-to-variable ratio of this dataset. External validation on the held-out test set (*n* = 30) yielded an accuracy of 83.3% (error rate 10.7%, unassigned 6.0%), with per-class metrics remaining acceptable (HC: sensitivity 1.000, precision 0.929; BD: sensitivity 0.714, precision 1.000; MDD: sensitivity 0.875, precision 0.778). Misclassifications were again concentrated at the BD/MDD boundary, consistent with the known clinical overlap between these conditions.

#### 3.2.4. Variable Importance Profiling: Identification of Principal Discriminatory Drivers

**Figure 5:**
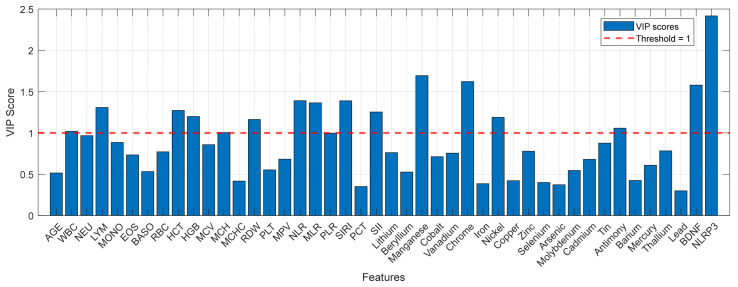
VIP scores derived from the three-class PLS-DA model (HC vs. MDD vs. BD) based on the autoscaled 42-biomarker matrix.

To identify which specific variables drive discrimination among the HC, BD, and MDD groups, we examined VIP scores from the PLS-DA model (Figure 5). Features with a VIP score greater than 1.0 are considered influential in the classification, while those exceeding 1.5 are deemed highly influential. The analysis revealed a hierarchical distribution of discriminatory biomarkers. The highest-ranking feature was NLRP3 (VIP > 2.0), followed by Manganese (Mn), Chromium (Cr), and BDNF (VIP > 1.5). These rankings are descriptive of their contribution to model separation and do not establish biological or mechanistic primacy. A broader set of features contributed at VIP > 1.0, including standard haematological indices and inflammatory ratios: LYM, HCT, HGB, NLR, MLR, SII, and SIRI, as well as the trace elements Nickel (Ni) and Antimony (Sb). It should be noted that in the MDD vs. BD binary model, lithium (VIP = 2.18) ranked among the highest features. Since lithium is a prescribed pharmacological agent predominantly used in BD, its discriminatory contribution reflects an iatrogenic effect rather than a primary pathophysiological difference between the two disorders; this confound is discussed in Section 4.7. Age also appeared as a discriminatory variable in the MDD vs. BD comparison (VIP > 1.0), reflecting the statistically significant demographic difference between cohorts rather than a disease-specific biological marker. Variables with VIP > 1.0 were interpreted as contributors to within-sample group discrimination, in line with the exploratory objective of the study.

**Figure 6:**
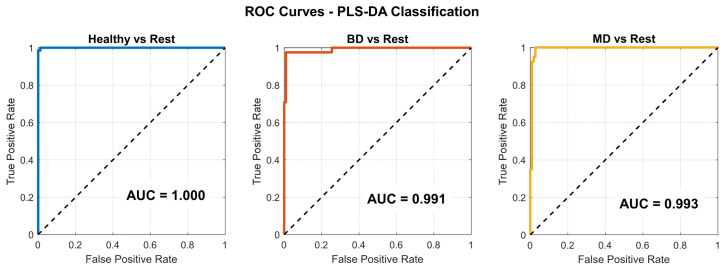
ROC curves derived from binary PLS-DA models, shown for descriptive purposes only within an exploratory framework. Each panel represents a one-vs-rest comparison (Healthy vs. Rest, BD vs. Rest, MD vs. Rest). Colored lines indicate the ROC curves for each model, while the dashed diagonal line represents the line of no discrimination (AUC = 0.5).

**Figure 7:**
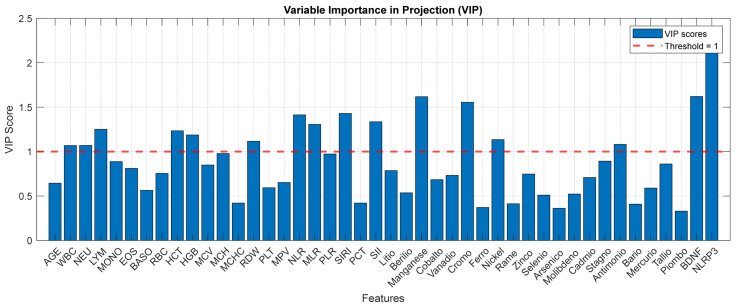
VIP scores derived from the binary PLS-DA model for the MDD vs. BD differential comparison, based on the autoscaled 42-biomarker matrix.

To identify the specific biomarkers distinguishing each clinical pair, we performed binary PLS-DA analyses for the three comparisons of interest: HC vs. MDD, HC vs. BD, and MDD vs. BD. For each pairwise comparison, models were constructed with a single latent component. Consistent with the three-class analysis, all variables were mean-centered and scaled to unit variance prior to modeling to ensure equal influence regardless of measurement units. A Bayesian decision rule was applied for class assignment, and model performance was evaluated using stratified 5-fold cross-validation, consistent with the procedure described in Section 2.6.2. Given the robust classification performance observed across comparisons, further discussion of predictive metrics is not central to the present investigation. Instead, attention is directed toward the VIP scores, which offer insight into the relative contribution of each biomarker to the discrimination between specific diagnostic pairs. The VIP scores from each model, which identify the biomarkers most influential in each pairwise discrimination, are presented as a heatmap in Figure 6 and summarized below. In the HC vs. MDD comparison, the biomarkers with the highest discriminatory power (VIP > 1.5) were NLRP3, BDNF, Nickel, Chrome, SII, SIRI, MLR and NLR. Another set of features with VIP scores exceeding 1.0 included WBC, NEU, LYM, RDW. PLR, Lithium, Manganese and Antimony. For the HC vs. BD comparison, a similar but distinct pattern emerged. NLRP3 again showed the highest VIP score, followed by Manganese (>2.0), Chromium, HCT, Molybdenum, HCT and HGB (VIP > 1.5). Additional influential biomarkers (VIP > 1.0) included WBC, RBC, MCH, RDW and Lithium. In the most clinically relevant comparison, MDD vs. BD, the biomarker profile was more selective (Figure 7). NLRP3 and BDNF are the dominant discriminators (VIP > 2.5), followed by LYM, MLR (VIP > 1.5) and AGE, EOS, HCT, HGB, NLR, PLR, SIRI, Manganese, Vanadium and Antimony. Notably, some routine hematological indices (WBC, NEU) fell below the VIP > 1.0 threshold in this differential diagnosis model, suggesting that while these markers effectively distinguish patients from controls, they are less capable of distinguishing between the two mood disorders. However, the trace elements Vanadium and Antimony retained VIP scores above 1.0 in the MDD vs. BD comparison.

**Figure 8:**
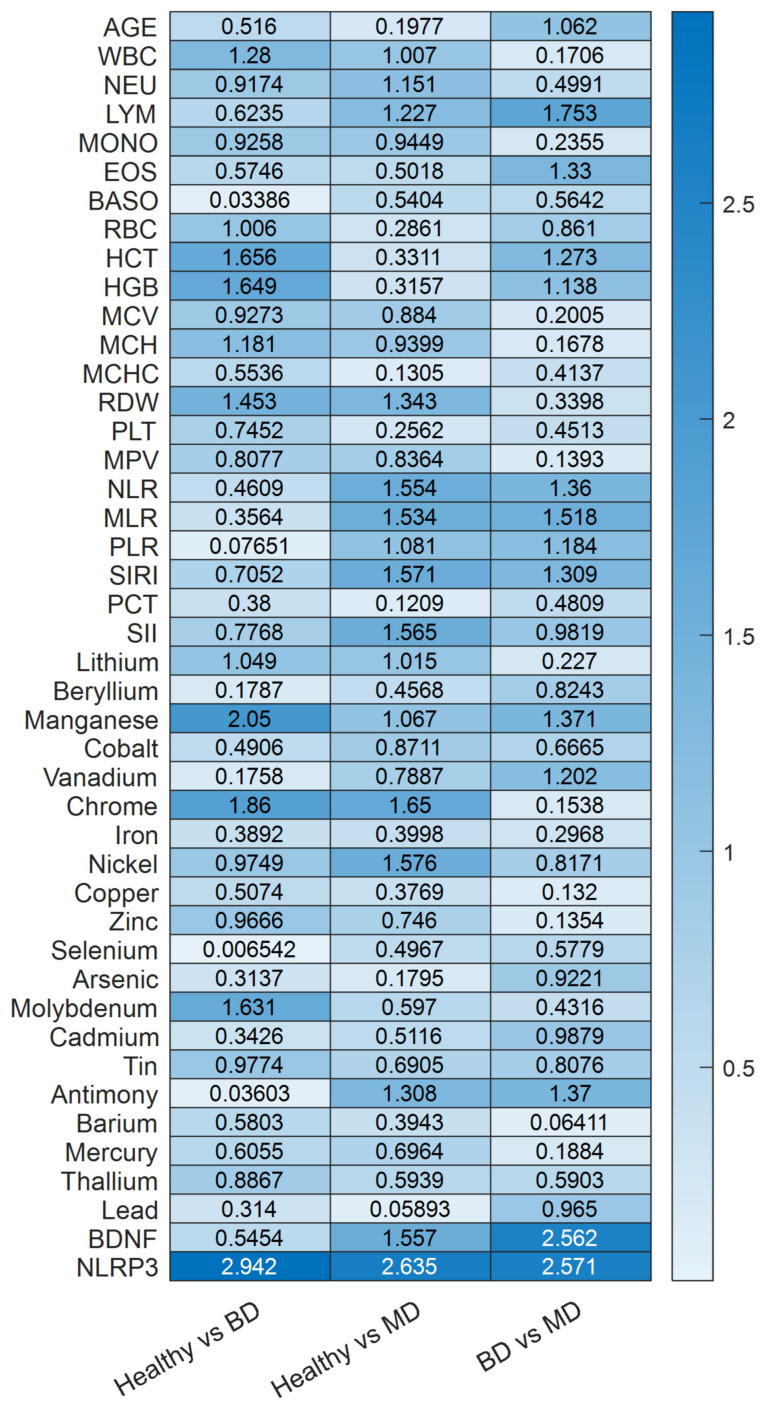
Heatmap representation of VIP scores derived from PLS-DA models across pairwise diagnostic comparisons (HC vs. BD, Healthy vs. MDD, BD vs. MDD). Darker color intensity reflects higher discriminatory contribution. NLRP3 and BDNF emerge as consistently high-ranking features across comparisons, supporting their transdiagnostic relevance.

The central intersection identifies NLRP3 and Manganese as consistent transdiagnostic features. These variables contributed across all pairwise comparisons, suggesting a shared peripheral signal. Partially overlapping zones reveal biologically coherent patterns: LYM, NLR, MLR, PLR, SIRI, BDNF, and Antimony are shared between HC vs. MDD and MDD vs. BD comparisons, underscoring the centrality of inflammatory imbalance and neurotrophic dysregulation in MDD-specific discrimination; HGB and HCT appear at the HC vs. BD/MDD vs. BD intersection, suggesting erythrocytic alterations as a preferentially bipolar feature. Chromium, WBC, NEU, RDW, and Lithium discriminate both psychiatric groups from controls, with Lithium interpreted as a pharmacological confound rather than a primary biological marker. Comparison-specific features reflect disorder-associated or demographic particularities: Nickel and SII are unique to HC vs. MDD; MONO, MCH, and Molybdenum to HC vs. BD; Age, EOS, Vanadium, and Cadmium to MDD vs. BD, with Age reflecting the significant demographic difference between cohorts rather than a pathophysiological determinant (Figure 8).

**Figure 9:**
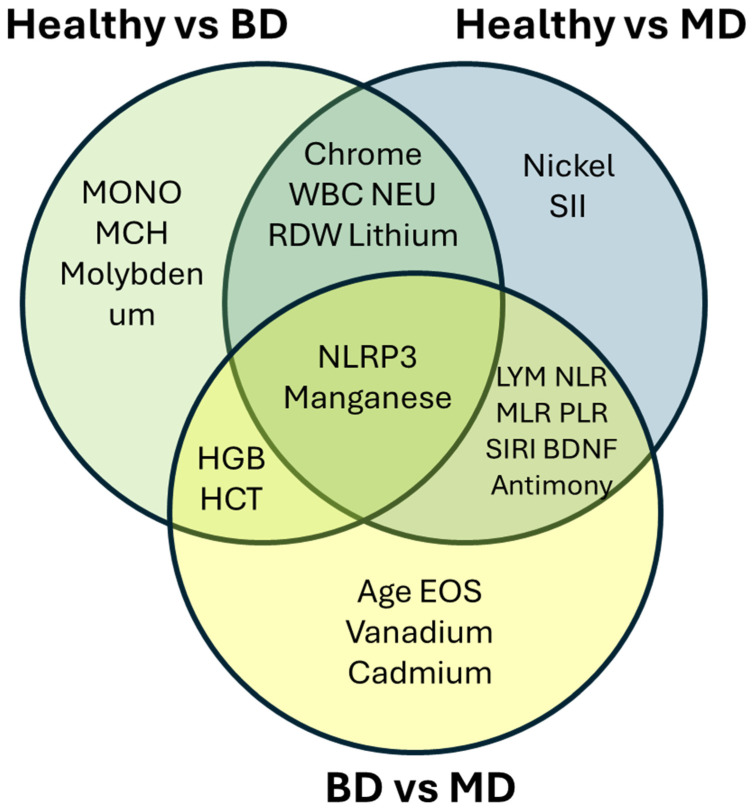
Venn diagram visualization of consistently discriminatory biomarkers across multiple pairwise comparisons, identifying transdiagnostic core biosignatures. Each colored circle represents a pairwise comparison (Healthy vs. BD, Healthy vs. MD, and BD vs. MD), while overlapping regions indicate biomarkers shared across comparisons.

Overall, the diagram, Figure 9, reveals a hierarchical biosignature architecture—a compact transdiagnostic core surrounded by partially shared and diagnosis-specific feature clusters—consistent with both common and divergent peripheral biological mechanisms underlying MDD and BD.

## 4. Discussion

This cross-sectional investigation indicates that integrated univariate and multivariate analytical approaches can identify coordinated peripheral biomarker patterns associated with Major Depressive Disorder and Bipolar Disorder. Convergent findings across analytical methods suggest the presence of multidimensional biochemical variation between diagnostic groups. However, all results must be interpreted as strictly associative and hypothesis-generating. No causal, mechanistic, or predictive inference is warranted from the present data. The observed between-group differences may reflect disease-related biology, pharmacological effects, demographic differences, or their interaction; these sources cannot be disambiguated within a cross-sectional framework.

### 4.1. NLRP3 Inflammasome Suppression

A notable observation was the marked reduction in circulating NLRP3 protein concentrations in both psychiatric cohorts relative to HC. This pattern is consistent with previous findings but does not establish a mechanistic relationship. An important analytical consideration must be noted: given that 61.4% of HC samples required serial dilution and back-calculation (see Section 2.4), the higher HC values carry greater quantitative uncertainty than those of the psychiatric groups, which fell within the assay’s calibrated range. Furthermore, circulating NLRP3 protein concentrations in plasma do not directly reflect intracellular inflammasome assembly or activation status; transcript-level and protein-level measurements in different compartments are not directly comparable. Several non-mutually exclusive interpretations may account for the observed pattern, none of which can be established from the present cross-sectional data: (i) reduced extracellular release relative to intracellular retention; (ii) altered protein stability or degradation kinetics; (iii) pharmacological modulation; or (iv) assay-related differences at high analyte concentrations. Meta-analytic evidence has shown that inflammatory dysregulation is a reproducible but non-specific feature of mood disorders, supporting the interpretation of these findings within a broader, non-diagnostic framework [18,94]. Future orthogonal validation strategies—including intracellular protein quantification by flow cytometry, functional caspase-1 activity assays, and IL-1β/IL-18 secretion analyses—are necessary before biological interpretation can be advanced. These findings should not be interpreted as direct evidence of inflammasome activation or functional activity.

The coexistence of elevated composite inflammatory indices (NLR, SIRI, SII) with reduced circulating NLRP3 concentrations is an associative observation that does not allow conclusions regarding underlying pathway activation. These two findings may or may not reflect related biological processes; inflammasome-independent inflammatory pathways, compartment-specific effects (e.g., CNS-resident microglia not sampled peripherally), or independent pharmacological effects are all plausible contextual factors. Mechanistic clarification requires prospective, longitudinal, and functionally validated investigations that are beyond the scope of the present study.

### 4.2. Composite Inflammatory Indices

Composite inflammatory ratios demonstrated consistent inter-group differences. Elevated NLR and SIRI values in MDD relative to BD and HC are associatively consistent with prior meta-analytic evidence linking low-grade systemic inflammation to depressive symptomatology. These indices integrate leukocyte subpopulations into summary metrics and may capture broader immune balance rather than isolated cell count fluctuations. Nevertheless, inflammatory alterations may represent state-dependent correlates, stress-mediated physiological responses, pharmacological effects, or demographic influences rather than disease-specific findings. Whether these patterns precede, co-occur with, or follow the onset of mood episodes cannot be determined from cross-sectional data. Longitudinal designs and causal inference approaches, including Mendelian randomization, are necessary to clarify directionality [95,96,97].

### 4.3. Metallome Perturbations: Statistical vs. Clinical Relevance

Alterations in trace element profiles were observed, including manganese elevation and zinc reduction in psychiatric groups. Importantly, statistical significance does not necessarily imply clinical relevance.

The magnitude of zinc reduction (~10% compared with controls) falls within reported intra-individual biological variation (coefficient of variation approximately 12%), raising the possibility that part of the observed difference reflects physiological fluctuation rather than pathophysiological depletion. Similarly, manganese concentrations in BD approached, but did not exceed, commonly cited neurotoxicity thresholds (>3–4 μg/L), suggesting subclinical accumulation without overt toxicological concern at present levels. These considerations underscore the distinction between detectability and actionability. Metallomic findings warrant cautious interpretation pending demonstration of functional consequences through longitudinal monitoring or interventional studies.

### 4.4. BDNF Biphasic Modulation

The differential pattern of BDNF—lower concentrations in MDD and higher concentrations in BD compared with HC—emerged as a discriminatory feature in both univariate and multivariate analyses. This pattern is associatively consistent with prior literature reporting reduced peripheral BDNF in depression and variable BDNF levels in bipolar disorder across mood states. However, the present data do not establish a causal or mechanistic relationship. In the BD group specifically, elevated BDNF concentrations may reflect pharmacological effects rather than primary disease biology: lithium and valproate are known to upregulate BDNF expression, and both agents are commonly prescribed in this cohort. Interpretation of BDNF as a pathophysiological rather than treatment-related marker in BD therefore requires caution. The biomarker profiles reported here suggest a preliminary biological stratification between groups. Nonetheless, the potential influence of pharmacological treatments, including lithium, should be considered as a contributing factor when interpreting these findings. Longitudinal assessments across mood state transitions and in medication-naïve cohorts are needed to clarify whether BDNF behaves as a state marker, trait marker, or treatment-responsive parameter. The simultaneous emergence of BDNF and NLRP3 as high-ranking variables in multivariate analysis is consistent with a biologically plausible reciprocal regulatory axis.

### 4.5. Methodological Considerations

The multivariate chemometric framework enabled integration of heterogeneous biological domains while accounting for multicollinearity. However, several methodological constraints must be emphasized. First, the sample-to-variable ratio of approximately 3.6:1 (151 observations, 42 variables) is at the lower boundary of recommended thresholds for PLS-DA modeling and increases susceptibility to overfitting. The Q^2^/R^2^Y diagnostic reported for each model provides a direct overfitting index: where Q^2^ closely approaches R^2^Y the model is considered stable; where the gap exceeds 0.2–0.3, results should be treated with additional caution. Permutation testing (1000 permutations) confirmed that observed classification performance exceeded chance-level distributions, but this does not substitute for external validation. All validation in the present study is internal, including stratified 5-fold cross-validation and an internal hold-out split. Internal cross-validation does not replace validation in an independent, prospectively recruited cohort; it provides an estimate of within-sample generalization only. Without external validation, the reported accuracy figures represent upper-bound estimates and cannot be assumed to generalize to other clinical populations, laboratories, or acquisition platforms. External validation in a demographically independent, multi-site cohort is designated as the primary objective of the planned follow-up study. Third, the observed inter-assay coefficients of variation (11.3% for NLRP3 and 9.1% for BDNF) approach desirable analytical performance targets derived from biological variation estimates; longitudinal within-subject variability assessment in larger cohorts would refine reference change value calculations and determine suitability for monitoring applications.

### 4.6. Future Directions

The primary next step for this research program is prospective external validation of the identified biosignature in an independent, demographically diverse, multi-site cohort. Such validation is essential before any translational relevance can be assessed. Translational implementation toward clinical biomarker qualification would further necessitate alignment with FDA/EMA regulatory frameworks specifying: (i) context-of-use definition (diagnostic adjudication vs. treatment response prediction vs. prognostic stratification); (ii) analytical validation demonstrating precision (CV < 15%), accuracy (recovery 90–110%), and linearity (r^2^ > 0.99) across physiological concentration ranges; (iii) clinical validation roadmaps with adequately powered prospective cohorts (estimated *n* = 600–800 per arm for 90% power, 5% type I error, AUC = 0.95 vs. null 0.50); (iv) clinical utility demonstration via decision curve analysis quantifying net benefit relative to treat-all/treat-none strategies, net reclassification improvement, and health economic cost-effectiveness modeling. The multidimensional analytical framework articulated herein represents a scalable platform compatible with high-throughput clinical laboratory infrastructure (estimated assay cost $150–200 per patient given multiplexed immunoassay + ICP-MS economies of scale), contingent upon demonstration of reproducibility (inter-laboratory CV < 20%) and clinical validity metrics in prospective evaluation aligned with biomarker qualification guidance [98].

### 4.7. Limitations

Several considerations circumscribe interpretive confidence. The cross-sectional design precludes causality inference, with observed perturbations potentially representing etiological contributors, state markers, or secondary consequences—disambiguable only through prospective longitudinal cohorts incorporating pre-morbid assessment and serial measurements across mood state transitions. Medication confounding in clinically ascertained cohorts complicates intrinsic pathophysiology attribution, although naturalistic treatment enhances ecological validity; medication-naïve first-episode cohorts would provide complementary drug-independent perspectives. Pharmacological confounding warrants explicit acknowledgment: lithium, which ranked among the highest VIP features in the MDD vs. BD differential model (VIP = 2.18), is prescribed predominantly in BD and must be interpreted as a potential iatrogenic discriminator rather than a primary pathophysiological biomarker. A sensitivity PLS-DA model with lithium excluded from the predictor matrix yielded accuracy of 89.4% for the three-class model and 89.4% for MDD vs. BD—a modest reduction that does not fundamentally alter the pattern of separation but confirms that lithium contributes to metallomic discrimination as a pharmacological confound. Pharmacotherapy class distribution across diagnostic groups is provided in Supplementary Table S2. Sample size (*n* = 151) proved adequate for exploratory discovery but independent validation in larger (target *n* > 500), demographically diverse, multi-site cohorts remains essential. Sex distribution heterogeneity (MDD: 82.9% female; BD: 57.5% male; HC: 64.3% female) and statistically significant age differences between cohorts (*p* = 0.004) represent potential confounds, as both sex and age modulate inflammation, BDNF, and trace element metabolism. ANCOVA analyses adjusted for sex and age were performed for the five primary biomarkers (NLRP3, BDNF, NLR, SIRI, manganese); inter-group differences remained statistically significant after adjustment, supporting the robustness of the core findings. Nevertheless, sex-stratified subgroup analyses were precluded by insufficient statistical power in the male MDD subgroup (*n* = 7). The FDR-corrected statistical analysis (Benjamini–Hochberg procedure) reduced the number of nominally significant findings from 37 to 34, with three borderline parameters (cobalt, mercury, beryllium) reclassified as nominally significant pending replication. Peripheral blood-derived analyte reliance introduces uncertainty regarding peripheral-central correlation; blood–brain barrier transport kinetics and compartment-specific regulation may decouple peripheral biosignatures from CNS pathophysiology. Finally, the absence of external validation means that all reported performance metrics should be treated as internally estimated upper bounds, not generalizable accuracy figures. Importantly, pharmacological treatment could not be withheld for ethical reasons, and therefore reflects real-world clinical conditions rather than experimental control. Consequently, biomarker patterns should be interpreted within a naturalistic clinical framework.

## 5. Conclusions

In summary, this study identified coordinated alterations in inflammatory indices, trace element profiles, and neurotrophic markers across mood disorder groups within a cross-sectional observational design. Multivariate modeling supported the presence of structured peripheral biomarker variation consistent with group differences; however, all findings are strictly associative. Causality, temporal stability, and clinical applicability remain entirely undetermined. The internally cross-validated classification performance should not be interpreted as clinically applicable classification performance; external validation in independent cohorts is required before any such interpretation is warranted. Prospective longitudinal studies, independent replication with FDR-controlled analyses, and analytical validation aligned with biological variation principles are required before consideration of clinical implementation. At present, these findings contribute to the exploratory characterization of peripheral multidomain profiling approaches in translational psychiatric research, and should be interpreted as hypothesis-generating only.

## Figures and Tables

**Table 8:** (Training Set Performance): Confusion matrix and performance metrics for the three-class PLS-DA model.

True Group	Predicted HC	Predicted BD	Predicted MDD	Not Assigned
**HC**	53	0	2	1
**BD**	0	28	1	3
**MDD**	0	1	30	2
**Overall metrics**	**Value**		**Per-class metrics**	**Value**
Accuracy	96.5%		*Precision*	
Error Rate (ER)	3.4%		HC	1.000
Not assigned	0.0%		BD	0.966
			MDD	0.909
			*Sensitivity*	
			HC	0.964
			BD	0.966
			MDD	0.968
			*Specificity*	
			HC	1.000
			BD	0.988
			MDD	0.964

**Table 9:** Internal Hold-Out Validation Set Performance: Confusion matrix and performance metrics for the three-class PLS-DA model.

True Group	Predicted HC	Predicted BD	Predicted MDD	Not Assigned
**HC**	13	0	0	1
**BD**	0	5	2	1
**MDD**	1	0	7	0
**Overall metrics**	**Value**		**Per-class metrics**	**Value**
Accuracy	83.3%		*Precision*	
Error Rate (ER)	10.7%		HC	0.929
Not assigned	6.0%		BD	1.000
			MDD	0.778
			*Sensitivity*	
			HC	1.000
			BD	0.714
			MDD	0.875
			*Specificity*	
			HC	0.933
			BD	1.000
			MDD	0.900

## Data Availability

The data supporting the findings of this study are available from the corresponding author upon reasonable request. Due to ethical and privacy restrictions related to the inclusion of human participants, the data are not publicly available.

## Supplementary Materials

The following supporting information can be downloaded at: https://www.mdpi.com/article/10.3390/medicina62050806/s1, Table S1: ANCOVA-adjusted biomarker analysis; Table S2: Pharmacotherapy Class Distribution Across Diagnostic Groups. Figure S1: additional multivariate visualizations; Figure S2: Permutation test results for the PLS-DA model.

## Abbreviations

AUC-ROC	Area Under Receiver Operating Characteristic Curve
BASO	Basophils
BD	Bipolar Disorder
BDNF	Brain-Derived Neurotrophic Factor
CCV	Continuous Calibration Verification
CNS	Central Nervous System
CV	Coefficient of Variation
DSM-5	Diagnostic and Statistical Manual of Mental Disorders, 5th Ed.
EDTA	Ethylenediaminetetraacetic acid
EMA	European Medicines Agency
EOS	Eosinophils
FDA	Food and Drug Administration
FDR	False Discovery Rate
HC	Healthy Controls
HCT	Hematocrit
HDRS-17	17-item Hamilton Depression Rating Scale
HGB	Hemoglobin
ICD-11	International Classification of Diseases, 11th Revision
ICP-MS	Inductively Coupled Plasma—Mass Spectrometry
ICV	Initial Calibration Verification
LQD	Limit of Quantification
LYM	Lymphocytes
MCH	Mean Corpuscular Hemoglobin
MCHC	Mean Corpuscular Hemoglobin Concentration
MCV	Mean Corpuscular Volume
MDD	Major Depressive Disorder
MLR	Monocyte-to-Lymphocyte Ratio
MONO	Monocytes
MPV	Mean Platelet Volume
NEU	Neutrophils
NLR	Neutrophil-to-Lymphocyte Ratio
NLRP3	NOD-like receptor pyrin domain-containing protein 3
PLS-DA	Partial Least Squares Discriminant Analysis
PCA	Principal Component Analysis
PLR	Platelet-to-Lymphocyte Ratio
PLT	Platelet Count
RBC	Red Blood Cell Count
RDW	Red Cell Distribution Width
RMSECV	Root Mean Square Error of Cross-Validation
ROC	Receiver Operating Characteristic
SCID-5	Structured Clinical Interview for DSM-5 Disorders
SIRI	Systemic Inflammatory Response Index
SII	Systemic Immune-inflammation Index
t-SNE	t-distributed Stochastic Neighbor Embedding
VIP	Variable Importance in Projection
YMRS	Young Mania Rating Scale
WBC	White Blood Cell Count
