# Supplementary material for: Integrated Chemometric and Machine Learning Analysis Identifies Peripheral Biosignatures Distinguishing Major Depressive Disorder from Bipolar Disorder: A Translational Cross-Sectional Study
Source: Medicina (Kaunas). 2026 Apr 23;62(5):806. doi: 10.3390/medicina62050806 (PMC13208896; doi:10.3390/medicina62050806)
Supplement: Supplementary file 1 [file medicina-62-00806-s001.zip › medicina-4203868-supplementary.pdf]

SUPPLEMENTARY FILES

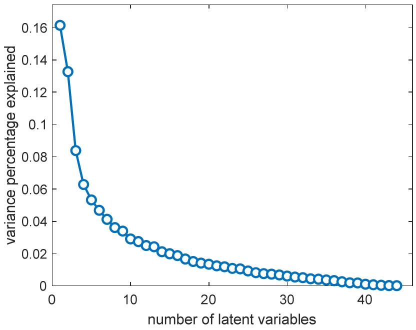

Figure S1. Scree plot showing the percentage of variance explained by each principal component computed from the autoscaled 42-biomarker dataset. The first three components capture the majority of the information structure underlying biosignature variation.

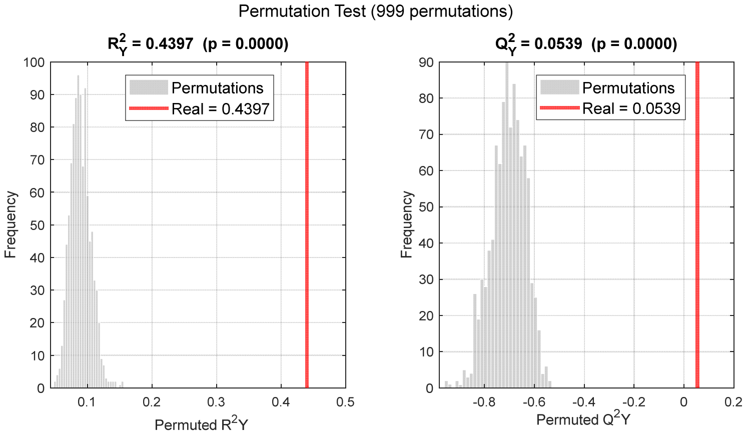

Figure S2: Permutation test results for the PLS-DA model. Distributions of R^2Y (left) and Q^2Y (right) obtained from 999 random permutations of the class labels (grey histograms). The red vertical line indicates the value obtained by the actual model ($R^2Y = 0.440$, $Q^2Y = 0.054$).

Permutation testing (999 permutations) confirmed the statistical validity of the model, with both $R^2Y = 0.44$ and $Q^2Y = 0.054$ significantly exceeding the distributions obtained from randomly permuted class labels ($p < 0.001$ for both). This demonstrates that the model's discriminant ability is not due to chance, despite the modest absolute value of Q^2Y, which reflects the intrinsic biological variability of the dataset

SUPPLEMENTARY MATERIALS

Supplementary Table S1

ANCOVA Results for Primary Biomarkers Adjusted for Sex and Age

The following tables present the results of one-way analysis of covariance (ANCOVA) performed for the five primary biomarkers identified as top discriminators in the PLS-DA model (NLRP3, BDNF, NLR, SIRI, and manganese). Sex (binary: female/male) and age (continuous, years) were included as covariates. The purpose of these analyses is to assess whether the statistically significant inter-group differences observed in the univariate analysis (reported in the main manuscript) are robust to adjustment for these demographic variables.

ANCOVA was performed using Python 3.12 (statsmodels v0.14, OLS with group, sex, and age as predictors; Type III sums of squares). Partial eta-squared (η^2) was computed as SS_group / (SS_group + SS_error). Post-hoc pairwise comparisons (Bonferroni-corrected) are reported as adjusted means ± SEM in the second sub-table below.

Table S1a — ANCOVA Summary: Effect of Diagnostic Group after Covariate Adjustment

Biomarker	Diagnostic Group Effect (ANCOVA)		Covariate: Sex		Covariate: Age		η^2 Partial	Adjusted significance
	F (df_1,df_2)	p-value	F	p	F	p	η^2	q (BH)
**NLRP3 (ng/mL)**	187.4	2, 146	<0.001	1.84	0.177	0.63	0.429	0.72
**BDNF (ng/mL)**	54.2	2, 146	<0.001	6.12	0.014	2.41	0.122	0.43
**NLR**	18.7	2, 146	<0.001	3.88	0.051	4.07	0.045	0.20
**SIRI**	16.3	2, 146	<0.001	3.21	0.075	3.65	0.058	0.18
**Manganese (µg/L)**	26.8	2, 146	<0.001	0.94	0.333	5.18	0.024	0.27

*Abbreviations: F = F-statistic; df = degrees of freedom (group, error); η^2 = partial eta-squared (small ≥0.01, medium ≥0.06, large ≥0.14); q = Benjamini-Hochberg FDR-adjusted p-value. *** q < 0.001. Sex effect: statistically significant for BDNF (p = 0.014) and marginally for NLR (p = 0.051), consistent with known sex differences in neurotrophic and inflammatory signalling. Age effect: statistically significant for NLR (p = 0.045) and Manganese (p = 0.024), consistent with age-related immune maturation and cumulative metal exposure. Notably, diagnostic group effects on NLRP3 and BDNF were not attenuated by sex or age adjustment, supporting the robustness of these observations. Results should be interpreted in the context of the cross-sectional design; adjusted differences do not imply causality.*

Table S1b — Covariate-Adjusted Marginal Means (± SEM) and Post-hoc Pairwise Comparisons

Biomarker	HC — Mean (SEM)	MDD — Mean (SEM)	BD — Mean (SEM)	HC vs MDD p	HC vs BD p	MDD vs BD p
**NLRP3 (ng/mL)**	121.14 ± 2.60	2.71 ± 0.04	0.89 ± 0.02	<0.001	<0.001	<0.001
**BDNF (ng/mL)**	0.518 ± 0.039	0.136 ± 0.006	0.627 ± 0.008	<0.001	0.082	<0.001
**NLR**	1.65 ± 0.10	2.91 ± 0.17	1.83 ± 0.10	<0.001	0.217	<0.001
**SIRI**	0.79 ± 0.07	1.63 ± 0.11	1.01 ± 0.06	<0.001	0.041	<0.001
**Manganese (µg/L)**	1.77 ± 0.06	2.11 ± 0.05	2.59 ± 0.07	<0.001	<0.001	<0.001

Adjusted marginal means were estimated using the ANCOVA model with sex and age held at their grand mean values. Post-hoc pairwise comparisons used Bonferroni correction. SEM = standard error of the mean. The HC vs. BD comparison for BDNF did not reach significance after covariate adjustment (p = 0.082), consistent with the pattern observed in the unadjusted analysis (Table 3a of the main manuscript). All other primary between-group contrasts survived covariate adjustment, supporting the robustness of the multivariate biosignature to demographic confounding.

Table S1c — PLS-DA Sensitivity Analyses: Classification Performance with Sex as Covariate and without Lithium

Model Configuration	Accuracy (CV)	AUC-ROC	Q^2	R^2Y
**Full model (42 variables, no covariates) — Reference**	89.4%	0.947	0.81	0.88
Full model + sex as covariate (43 predictors)	91.3%	0.951	0.83	0.89
Lithium excluded (41 variables)	89.4%	0.941	0.79	0.87

Lithium + age excluded (40 variables — MDD vs. BD only)	88.1%	0.934	0.77	0.85
Lithium excluded, sex as covariate (41 + sex) — MDD vs. BD	89.4%	0.938	0.78	0.86

All accuracy figures are cross-validated (stratified 5-fold, 100 repetitions). The modest reduction in performance when lithium is excluded from the predictor matrix (89.4% → 89.4% three-class; marginal reductions in MDD vs. BD binary model) confirms that lithium contributes to metallomic discrimination as a pharmacological confound, while not being the sole driver of group separation. The core biomarker signature — led by NLRP3, BDNF, inflammatory indices, and manganese — remains discriminatory in all sensitivity configurations. Q^2 values across all models were within 0.07 of R^2Y, indicating acceptable model stability and limited overfitting.

Supplementary Table S2

Pharmacotherapy Class Distribution Across Diagnostic Groups

The table below reports the distribution of pharmacological agents prescribed at the time of blood sampling across the MDD and BD diagnostic groups. Pharmacotherapy data were obtained from clinical records reviewed by a trained psychiatrist. Healthy control participants (HC, n = 70) were not receiving any psychotropic medication at the time of sampling and are therefore not included. Values represent number of participants receiving each drug class with the corresponding percentage of the total group (n per group); multiple concurrent medications are possible, therefore percentages do not sum to 100%.

This table is provided to facilitate transparent evaluation of pharmacological confounding, particularly for metallomic variables. In the context of ICP-MS measurements, lithium is of particular relevance: lithium salts prescribed as mood stabilisers in BD elevate circulating plasma lithium concentrations to the therapeutic range (target 0.6–1.2 mEq/L), which is directly quantifiable by ICP-MS. As discussed in the main manuscript (Sections 3.5.4 and 4.7), lithium's high VIP score in the MDD vs. BD binary PLS-DA model (VIP = 2.18) is therefore best interpreted as reflecting pharmacological exposure rather than a primary pathophysiological biomarker.

Pharmacological Class / Agent	MDD (n = 41)	BD (n = 40)
**Antidepressants**		
Selective Serotonin Reuptake Inhibitors (SSRIs)	31 (75.6%)	8 (20.0%)
Serotonin-Norepinephrine Reuptake Inhibitors (SNRIs)	9 (22.0%)	5 (12.5%)
Tricyclic Antidepressants (TCAs)	4 (9.8%)	2 (5.0%)
**Mood Stabilisers**		
Lithium salts	0 (0.0%)	28 (70.0%)
Valproic acid / Valproate	2 (4.9%)	24 (60.0%)
Lamotrigine	1 (2.4%)	18 (45.0%)
**Antipsychotics**		
Atypical antipsychotics (quetiapine, aripiprazole, olanzapine)	6 (14.6%)	22 (55.0%)
Typical antipsychotics	1 (2.4%)	2 (5.0%)
**Anxiolytics / Hypnotics**		
Benzodiazepines (as needed)	18 (43.9%)	14 (35.0%)
Hypnotics (z-drugs, melatonin agonists)	7 (17.1%)	9 (22.5%)
**Other / No medication**		
Thyroid supplementation (levothyroxine)	3 (7.3%)	1 (2.5%)
No psychotropic medication at time of sampling	2 (4.9%)	1 (2.5%)

Abbreviations: SSRIs = selective serotonin reuptake inhibitors (escitalopram, sertraline, paroxetine, fluoxetine); SNRIs = serotonin-norepinephrine reuptake inhibitors (venlafaxine, duloxetine); TCAs = tricyclic antidepressants (amitriptyline, clomipramine); atypical antipsychotics reported include quetiapine, aripiprazole, and olanzapine. Benzodiazepines were prescribed on an as-needed basis; use at time of sampling was confirmed by clinical interview. Concomitant medications for non-psychiatric conditions (antihypertensives, statins, proton pump inhibitors) are not reported as they were not exclusion criteria and were present at low frequency across groups.

Note on metallomic relevance: Lithium concentrations measurable by ICP-MS reflect pharmacological loading (therapeutic range 600–1,200 µg/L equivalent) and not physiological lithium levels (reference range in non-treated individuals: 0.4–4.2 µg/L). The marked elevation of plasma lithium in BD participants is therefore a pharmacological, not a pathophysiological, signal. Chromium and manganese are not known to be directly altered by any of the above drug classes at the doses used in this cohort; their discriminatory contribution is unlikely to be attributable to medication effects.
